# The Impact of the Amendments of the Medical Act 1971 [Act 50] in 2024 on the Implementation of Specialist Training Programmes by the Ministry of Health

**DOI:** 10.21315/mjms-02-2025-s01

**Published:** 2025-06-30

**Authors:** Hirman Ismail, Mohd Azman Yacob, Mohamed Hirman Abdullah, Ahmad Badruridzwanullah Zun, Sabrizan Osman, Siti Baizura Amran, Nur Ainina Idris, Farah Ruwaida Fakhrul Ruzi, Siti Norsyazwanis Jalaluddin, Badiuzzaman Abd Kadir, Faarhana Che Arshad, Nor Akmal Hakim Kamarulzaman

**Affiliations:** Medical Development Division, Ministry of Health, Putrajaya, Malaysia

**Keywords:** healthcare system, human resource for health, Medical Act 1971, medical education, medical specialty, medical subspecialty, Ministry of Health, training

## Abstract

Due to ongoing and unresolved disputes over the recognition of specialist training programmes and specialist registration, the Ministry of Health and the Ministry of Higher Education sought legal opinion from the Attorney General’s Chambers. Following the legal opinion, both Ministries proposed a joint memorandum to the Cabinet to amend the Medical Act 1971 [Act 50]. This article outlines the need for these amendments, their content, and their impact on specialist training programmes conducted by the Ministry of Health.

## Introduction

The Medical Act 1971 [Act 50] was enacted and came into force on 1 October 1971 to regulate the registration and practice of medical practitioners in Malaysia. However, when it was first enacted, there was no specific provision to recognise and register medical specialists among these registered medical practitioners. This oversight allowed medical practitioners to self-claim and practice as specialists. Such practices were misleading and could potentially affect patients’ safety. Therefore, in 2012, Act 50 was amended to introduce specialist registration through a specialist register, better known as the National Specialist Register (NSR), under the Malaysian Medical Council (MMC). This has allowed medical practitioners with recognised specialist qualifications to register and publicly declare themselves as legitimate specialists. The Medical (Amendment) Act 2012 [Act A1443] came into force on 1 July 2017, five years after it was passed by the Malaysian Parliament in 2012. Following the enforcement of Act A1443, the MMC adopted a list of recognised specialist qualifications, which was accessible through the MMC official website. Medical practitioners who hold any of the listed qualifications were entitled to be registered as specialists in the NSR.

## The Needs for the Amendments in 2024

The inability to register medical practitioners who have completed the Parallel Pathway Specialist Training Programmes conducted by the Ministry of Health (MOH), particularly in cardiothoracic surgery, has sparked numerous discussions and debates about the specialist registration process and recognition of specialist training programmes in the country. The qualification used in the Cardiothoracic Surgery Parallel Pathway Specialist Training Programme, Fellowship of the Royal College of Surgeons of Edinburgh (RCSEd) in Cardiothoracic Surgery, was said to be not listed as a recognised specialist qualification (1, 2). There have been conflicting opinions regarding the role of accreditation by the Malaysian Qualifications Agency (MQA) in the process of recognition of specialist training programmes by the MMC. This issue has left the status of some so-called unlisted international specialist qualifications used by the MOH in their parallel pathway specialist training remained unresolved. It is not so clear since when the issue regarding the recognition of the qualification was discussed or debated, but the Academy of Medicine of Malaysia, the RCSEd and the Malaysian Association of Thoracic and Cardiovascular Surgery have been advocating for the inception of the training programme by the MOH since at least 2014 when a memorandum of understanding was signed to develop a national training programme (3).

Four pioneering trainees were admitted into the programme in 2016, and between 2018 and 2022, several accreditation visits were conducted by the RCSEd, where eight centres were recognised as certified training centres for the programme (3). The training programme was presented and approved by a central committee chaired by the Director General of Health (DG) called Special Medical Committee or Jawatankuasa Khas Perubatan (JKP) in 2014, before the Medical (Amendment) Act 2012 [Act A1443] was enforced in 2017. JKP is a committee established under General Order 27 (Perintah 27 Bab F Perubatan Perintah-Perintah Am). JKP is further described in the later part of this article. The long-standing and unresolved discussions and debates with regard to the recognition of the qualifications have driven the MOH and the Ministry of Higher Education (MOHE) to consult the Attorney General’s Chambers (AGC) on this matter. The decision to consult AGC was made in a high-level meeting between both Ministries on 6 June 2023.

A legal opinion by the AGC was obtained by the MOH on 25 March 2024, and part of the opinion was explained by the Minister of Health during the second reading of the amendment bill (the draft amendment to the Medical Act 1971) in Dewan Rakyat (The House of Representatives the Malaysian Parliament) on 16 July 2024. The main issue regarding specialist registration under the Medical Act 1971 [Act 50] stems from the criteria of registration as listed in section 14B of Act 50. The provisions of section 14B of Act 50 stated that (4), in addition to holding a “recognised specialist qualification,” a person would be entitled to be registered as a medical specialist if the person has attended specialist training at any recognised training institution. The phrase “recognised training institution” was defined in section 2 of Act 50 as a “higher education provider” as defined under section 2 of the Malaysian Qualifications Agency Act 2007 [Act 679]. The cross-referencing for such definition means that a training institution recognised under Act 50 must fulfil the characteristics of a “higher education provider” as prescribed under Act 679. Therefore, based on the definition of a recognised training institution under Act 50, the MOH facilities could not be construed as recognised training institutions in the context of a “higher education provider” pursuant to the said provisions in these two acts [Act 50, Act 679].

Therefore, the Parallel Pathway Specialist Training Programmes and the Master of Medicine Specialist Training Programmes conducted by public universities at facilities owned by the MOH did not meet the criteria outlined in paragraph 14B(b) of Act 50. In most specialist training in both pathways, trainees are required to attend specialist training, especially the clinical rotations, in MOH facilities such as hospitals, health clinics and health district offices. Such interpretation has made both Parallel Pathway and the Master of Medicine Specialist Training Programme irregular from the legal point of view, and the eligibility of trainees to be registered as specialists under Act 50 was questionable (5, 6). Such irregularities required immediate rectification through amendments of Act 50 to save both specialist training pathways, given its potential legal implications to the practitioners, providers and the system as a whole (6, 7).

## The Process of the Amendments

Following the legal opinion by the AGC, both the Minister of Higher Education and the Minister of Health, in a meeting attended by both Ministers and senior officials on 18 April 2024, reached a consensus on the need for the amendment of the Medical Act 1971 [Act 50] to address issues of specialist training recognition and specialist registration (8). On 5 June 2024, a joint Cabinet paper was presented to the Cabinet by both Ministers, which led to approval for amending the act. Stakeholders’ engagements were essential to explain the legal opinion of AGC and to emphasise the need to rectify the issues through the amendments. Before early July 2024, at least 20 series of engagements were conducted on the proposed amendments with 62 stakeholders involving government agencies, professional bodies and nongovernmental organisations. Engagements with the Parliamentary Special Select Committee on Health were held on 4 and 10 July 2024. On top of that, there were a series of close discussions with the AGC office alongside the engagements.

The final amendment bill was submitted to the Cabinet through a memorandum by the Minister of Health on 5 July 2024 for tabling to the Parliament. The Medical (Amendment) 2024 Bill went through the first reading in Dewan Rakyat and the subsequent second reading and debate by Members of Dewan Rakyat. Before the debate, after the first reading in Dewan Rakyat, the Minister of Health held a briefing with Members of Dewan Rakyat to explain the proposed amendments. The draft amendment bill was available to all Members and the public for review. It generated significant interest, with 20 Members of Dewan Rakyat participated in the parliamentary debate during the second reading (5, 7). Following the debate and the third reading, the Dewan Rakyat approved the bill without further amendments on 17 July 2024.

**Figure 1:**
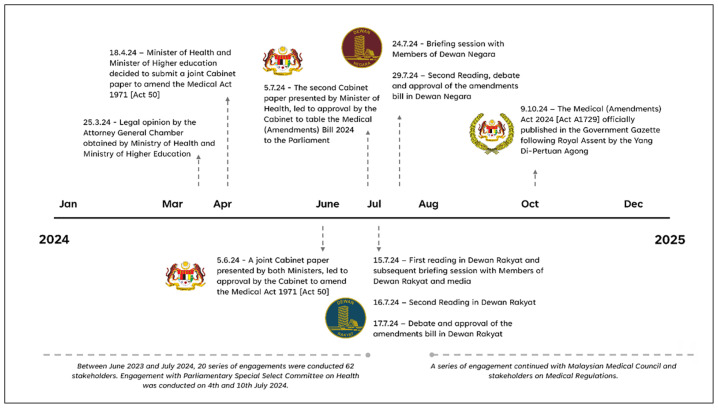
Timeline of the amendments of the Medical Act 1971 [Act 50] in 2024

**Figure 2:**
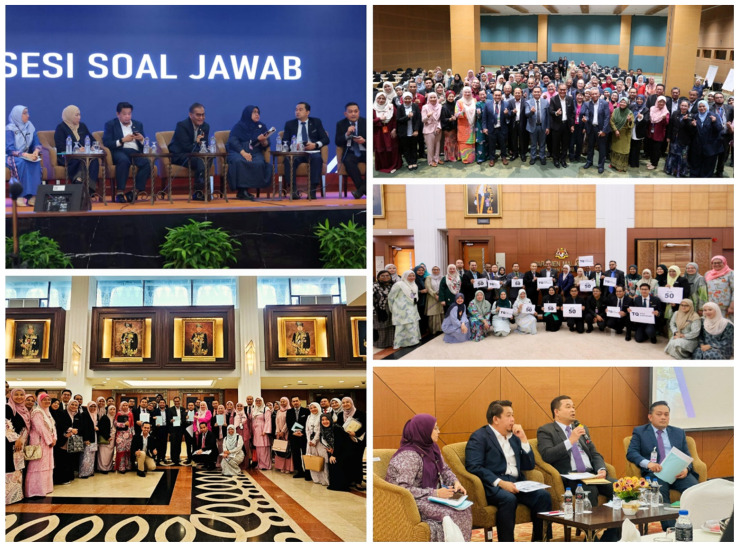
Process of amending the Medical Act 1971 [Act 50] (anti clockwise from top left): Picture 1, briefing session with Members of the Dewan Rakyat (Lower House of the Parliament) after the first reading of the Amendments Bill on 15 July 2024 led by the Minister of Health; Picture 2, group photo with the working group from MOH, MOHE and the AGC after the Amendments Bill was passed by the Dewan Rakyat on 17 July 2024; Picture 3, briefing session with Members of the Dewan Negara (Upper House of Parliament) on 24 July 2024 before the second reading of the Amendments Bill led by the Deputy Minister of Health; Picture 5, group photo with the working group from MOH, MOHE and the AGC after the Amendments Bill was passed by the Dewan Negara on 29 July 2024; Picture 6, group photo with Members of the MMC in an engagement session on 19 August 2024 after the Amendments Bill was passed by the Parliament.

Upon approval of the amendment bill in Dewan Rakyat, the subsequent second reading in the Dewan Negara (The Senate of the Malaysian Parliament) was held on 29 July 2024 with 14 Members of Dewan Negara or Senators participated in the debate (6). Prior to the second reading and debate, the Deputy Minister of Health hosted a luncheon on 24 July 2024 to brief and engage the Members of Dewan Negara on the proposed amendments. The amendment bill was approved by the Dewan Negara on the very same day of the second reading and debate without further amendment to the bill. The amendment bill received the Royal Assent from the Yang Di-Pertuan Agong (the King of Malaysia) on 9 October 2024, and subsequently, it was published in the Government Gazette as the Medical (Amendment) Act 2024 [Act A1729], on 17 October 2024, which was made available through the AGC’s Federal Legislative Portal, https://lom.agc.gov.my/. The timeline for the process of amending the Medical Act 1971 [Act 50] is illustrated in Figure 1 and Figure 2.

## The Contents of the Amendments

In general, seven sections of the Medical Act 1971 [Act 50] were amended, and one section was created as a new provision under the amended act known as the Medical (Amendment) Act 2024 [Act A1729] – section 2 on interpretation or definition of terms, section 3A on composition of the MMC, section 4A on power of the MMC, section 14 on full registration as medical practitioner, section 14B on the qualifications or criteria of specialist registration, section 14C on the process of specialist registration, section 14D, which was a new provision on registration of specialist in subspecialty areas and section 36 related to regulations under the law (5, 6, 9). Two new schedules were created under Act 50: Fourth Schedule on List of Registrable Specialist Qualifications and Fifth Schedule on List of Subspecialty. Saving and transitional provisions were included in section 11 of Act A1729 and validation in section 12 of Act A1729. A revised set of criteria for registration of specialists in the NSR is outlined in section 14B. There are five major criteria compared to the previous four criteria.

### Criteria 1

Only medical practitioners who are fully registered under Act 50 can be considered for registration as specialists (para 14B(1)(a)).

### Criteria 2

Registered medical practitioner (RMP) must also hold any of the specialist qualifications as specified in the fourth column of the Fourth Schedule for a duration of recognition that may be determined by the MMC (para 14B(1)(b)). The Fourth Schedule was created under the amended law to ensure proper governance of the qualifications list. With the new schedule, the listing of the qualifications is now more structured. The Fourth Schedule was constructed based on the structure of the existing Second Schedule on registrable undergraduate or primary medical qualifications. There are 296 qualifications listed in the Fourth Schedule, of which 115 qualifications originated from Malaysia, followed by United Kingdom (66), United States of America (36), Australia (30), Republic of Ireland (23), Canada (11) and others (15). Under subsection 14B(4), the MMC may amend the list from time to time as necessary, and the list can be perfected further.

### Criteria 3

Holding a qualification alone will not necessarily qualify an RMP to be registered as a specialist because, as specified in paragraph 14B(1)(c), the RMP must also demonstrate that he or she has completed specialised training. For example, someone who holds a certificate of Membership of the Royal College of Physicians of Ireland, must demonstrate that he or she has completed the necessary training such as, among others, clinical rotations, workplace-based assessment and evaluation through an institutionalised training programme in the country. These shall be fulfilled before he or she can be considered for registration as a specialist. In other words, holding a qualification certificate alone will not automatically qualify for registration unless the RMP has demonstrated, through relevant evidence, that he or she has undergone an institutionalised training programme. Paragraph 14B(3)(b) of the Act also provides power to the MMC to evaluate training programmes outside Malaysia.

### Criteria 4

On top of holding a qualification and having completed specialised training, an RMP must also demonstrate that he or she has undergone supervised work experience (SWE) as specified in subparagraph 14B(1)(d)(i). SWE is to streamline the process of probation of specialists who have completed specialist training, or a process also previously known as “specialist gazettement.” The gazettement process was specific to doctors working in the public sector. Before specialist registration was introduced through the amendments of Act 50 in 2012, the process was the only process within the public sector to formally recognise specialists. Under General Order 27 in Chapter F on Medicine of the General Orders (Perintah 27 Bab F Perubatan Perintah-Perintah Am), the DG, on the advice of three other specialists in the public sector or a committee known as Special Medical Committee or JKP, has the power to appoint any medical officers within the public sector as specialist after the DG has satisfied that the person holds relevant and recognised qualifications and possesses experience to the satisfaction of the DG. It was a process that has been practised in the public sector for many years to ensure that those who were appointed as specialists were competent and safe. It is not known when the specialist gazettement was initiated, but the oldest record of gazettement available under the keeping of the Medical Development Division was dated 7 September 1987. Chapter F on Medicine of the General Orders was approved by Yang Di-Pertuan Agong on 1 August 1957, with the latest version approved on 1 March 1974. The General Orders in Malaysia refer to a set of regulations and guidelines that govern the conduct, appointment, promotion, and other administrative aspects of public service employees. The process of specialist gazettement is to be streamlined with SWE following the amendments, while RMP, who are not public servants, shall be subjected to evaluation by MMC for the requirements of SWE. Subparagraph 14B(1)(d)(ii) allows the MMC to register an RMP as a specialist without having to go through the SWE if he or she has proven his or her work experience to the satisfaction of the MMC. This may apply to those who have vast work experience as specialists outside the country and are undergoing such probation or SWE, which may be superfluous.

### Criteria 5

Paragraph 14B(1)(e) also requires the RMP to be proven competent and of good character to qualify him or her to be registered as a specialist.

## The Impact of the Amendments

The amendments of the Medical Act 1971 [Act 50] have a significant impact on the implementation of specialist training programmes conducted by the MOH. There are 120 Master of Medicine Specialist Training Programmes conducted by the local higher educational institutions and 14 Parallel Pathway Specialist Training Programmes under the MOH. The MOH spent RM365.4 million on sponsorship for these training programmes between 2019 and 2023, of which 96% of that allocation were spent on Master of Medicine Specialist Training Programme (7). Since 2014, the Parallel Pathway Specialist Training Programmes have undergone a series of restructuring. This includes the establishment of a central governance body (Main Committee for Parallel Pathway Specialist Training and subcommittees by each specialty) to oversee the whole implementation of the programme. The structure of training, monitoring, assessment, duration, and entry criteria have been standardised, mirroring the Master of Medicine programmes. Such standardisation of implementation of Parallel Pathway Specialist Training Programmes shall be enhanced in the near future following the amendments of the Medical Act 1971 and following the move by MMC to develop Specialty Specific Requirements (SSR).

The SSR is a standard for specialist training programmes in the country that sets a benchmark on, among others, entry criteria, duration of training, structure of training, clinical rotations, assessment and list of competencies, where all these requirements shall be applied to both Master of Medicine Specialist Training Programmes and Parallel Pathway Specialist Training Programmes. At the time of writing, 23 SSR for various basic specialities have been approved and published by the MMC, and 6 SSR were still under development. Standardisation shall also consider the development of the National Postgraduate Medical Curriculum (NPMC), led by the Medical Deans’ Council of Public Universities, a collaborative initiative with multiple stakeholders, including experts from the MOH (10). The initiatives to ensure the standardisation of the implementation of the Parallel Pathway Specialist Training Programmes started as early as 2023 through a workshop organised by the Medical Development Division with all heads of training for all 14 training programmes, held between 8 and 12 July 2023, to develop a general training management guideline and guidelines specific to each specialty (Figure 3). In the workshop, focus was given to promote sustainable governance, standard structure of training and clinical rotations, methods of assessment and certification of completion of training, with the primary aim to ensure quality of training and patient safety. These guidelines shall be reviewed from time to time to ensure their consistency with the SSR and NPMC.

Through the amendments of the Medical Act 1971 [Act 50], as provided in paragraph 14B(3)(a), the MOH is now considered one of the local training providers in the country, apart from the local higher educational institutions. Such provision in the amended act has addressed the irregularities in the definition of recognised training institution as defined in section 2 of the original act as previously described above. Specialised training, as referred in the amended act, particularly in paragraph 14B(1)(c), shall be construed as a reference to, in the context of the local specialist training programme, a training approved by the MMC in relation to a specialty listed in the Fourth Schedule and provided by either the MOH, local higher educational institutions or any other institution in the country as approved by the MMC (9). The *locus standi* of the MOH and its facilities as training providers or training centres has been made regular and conformed from the legal point of view. Under subsection 11(9) of Act A1729, any training in a specialty before the enforcement of the amendments shall be construed as specialised training under the Act, and that includes the existing training provided by the MOH through the Parallel Pathway Specialist Training Programmes. The same subsection 11(9) of Act A1729 has also addressed the irregularities in the implementation of Master of Medicine Specialist Training Programme conducted at facilities owned by the MOH as pointed out by the AGC in its legal opinion as previously described above (5,6). All international specialist qualifications used in the existing MOH Parallel Pathway Training Programmes have been listed in the new Fourth Schedule as itemised in Table 1.

**Figure 4:**
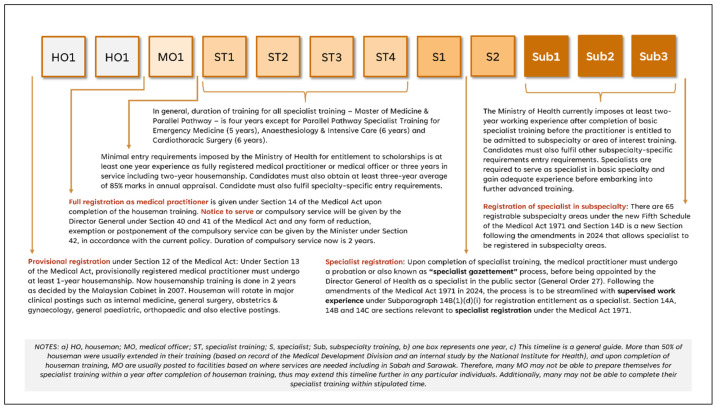
Relationship between the Medical Act 1971, including the amendments in 2024, with a medical practitioner’s career journey and experience under the MOH’s ecosystem

It is also important to enhance the training experience of all trainees, and that can be achieved by organising sustainable and regular supporting training activities such as preparatory courses, mock examinations, skill labs and compulsory training as required by the SSR. Trainers shall be engaged frequently, especially the educational supervisors and training of trainers shall be made available on a regular basis. The Medical Development Division has developed a central database of all trainers and trainees by each specialty and updated it by location in all training centres, through which a dashboard could be developed to visualise trainee-to-trainer ratios by each locality nationwide (Figure 3). Such monitoring shall be consistent to ensure trainers are appropriately guided and receive adequate exposure. This article may not discuss other aspects of the amendments, such as registration in subspecialty areas, recognition process of specialist training or registration of specialists with qualifications not listed in the Fourth Schedule under subsection 14B(2) of Act 50. Some of the impacts of the Medical Act 1971, including amendments in 2024 on a medical practitioner’s career journey and experience are illustrated in Figure 4.

## Conclusion

The Medical Act (Amendment) 2024 [Act A1729] officially came into force on 1 July 2025, following the endorsement of the Medical Regulations (Amendment) 2025. The Regulations were formally signed on 26 June 2025 by the Minister of Health and the Director-General of Health, who also serves as the President of the Malaysian Medical Council. The amendments of the Medical Act 1971 [Act 50] have a significant impact on the implementation of specialist training programmes in the country. Continuous enhancement of the Parallel Pathway Specialist Training Programme conducted by the MOH shall be made where efforts shall be focused to ensure sustainable governance and monitoring on the implementation of the training programme as previously discussed. The proposal to establish a unified national governing body that encompasses both Master of Medicine Specialist Training Programme and Parallel Pathway Specialist Training Programme involving multiple stakeholders in public and private sectors, as advocated by the Academy of Medicine Malaysia (11), shall be explored and studied on its feasibility. The interest in specialisation among the medical officers, particularly within the MOH, remains high (10), and number of trainees is expected to grow. The need for specialist training in the country to shift to Competency-Based Medical Education (CBME)(10) requires a long-term commitment to educational reform and may justify the need for such a unified national governing body. Such proposal can be part of intermediate or long-term strategic moves to address gaps between the needs and supply of doctors including specialists, particularly in the public sector as quantified and discussed in our previous editorials in this journal (12, 13).

## Figures and Tables

**Figure 3a:**
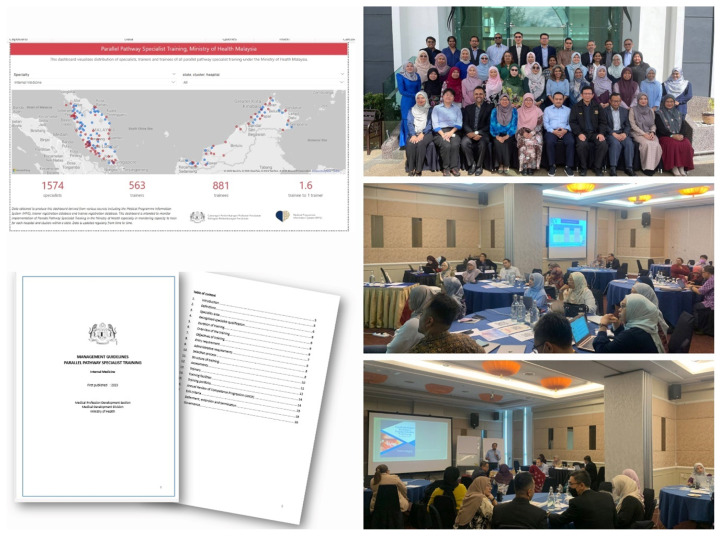
Standardising the implementation of Parallel Pathway Specialist Training Programme conducted by the MOH (anti clockwise from top left): Picture 1, dashboard to monitor the location of trainers and trainees, and trainee-to-trainer ratio; Picture 2, training management guideline specific to each specialty; Pictures 3–5, workshop organised by the Medical Development Division with all heads of training for all 14 training programmes (8–12 July 2023), to develop a general training management guideline and specialty specific guidelines

**Figure 3b:**
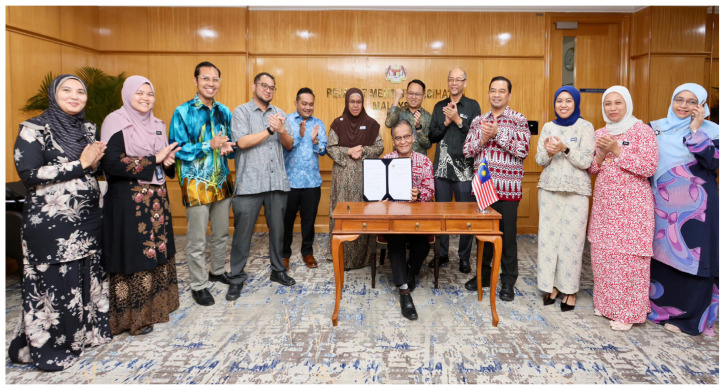
The signing of the Medical Regulations (Amendment) 2025, alongside the official enforcement date of the Medical Act (Amendment) 2024 [Act A1729]. The documents were signed by the Minister of Health on 26 June 2025 at the Ministry of Health headquarters in Putrajaya

**Table 1:** List of Parallel Pathway Specialist Training Programme conducted by the MOH and list of Specialist Qualifications in accordance with the Fourth Schedule of the Medical Act 1971 [Act 50]

No.	Specialty	Qualification	Awarding body	Trainees	Trainers
1.	Anaesthesiology and Critical Care	Fellowship of the College of Anaesthesiologists of Ireland *Listed as Registrable Specialist Qualification as item (1)(f)(i) in Fourth Schedule*	College of Anaesthesiologists of Ireland Country: Ireland	61	127
2.	Obstetrics and Gynaecology	Membership of the Royal College of Obstetricians and Gynaecologists *Listed as Registrable Specialist Qualification as item (5)(c)(i) in Fourth Schedule*	Royal College of Obstetricians and Gynaecologists of United Kingdom Country: United Kingdom	260	180
3.	Ophthalmology	Fellowship of the Royal College of Ophthalmologists *Listed as Registrable Specialist Qualification as item (6)(g)(ii) in Fourth Schedule*	Royal College of Ophthalmologists of United Kingdom Country: United Kingdom	53	81
4.	Oncology	Fellowship of the Royal College of Radiologists *Listed as Registrable Specialist Qualification as item (7)(c)(i) in Fourth Schedule*	Royal College of Radiologists of United Kingdom Country: United Kingdom	11	26
5.	Forensic Pathology	Fellowship of the Royal College of Pathologists of Australasia in Forensic Medicine *Listed as Registrable Specialist Qualification as item (12)(b)(i) in Fourth Schedule*	Royal College of Pathologists of Australasia Country: Australia	NA	NA
		Diploma in Medical Jurisprudence (Pathology) *Listed as Registrable Specialist Qualification as item (12)(c)(iv) in Fourth Schedule*	Society of Apothecaries, London, United Kingdom Country: United Kingdom	7	13
6.	General Paediatric	Membership of the Royal College of Paediatrics and Child Health *Listed as Registrable Specialist Qualification as item (15)(e)(i) in Fourth Schedule*	Royal College of Paediatrics and Child Health of United Kingdom Country: United Kingdom	286	517
7.	Cardiothoracic Surgery	Fellowship of the Royal College of Surgeons of Edinburgh in Cardiothoracic Surgery *Listed as Registrable Specialist Qualification as item (17)(e)(ii) in Fourth Schedule*	Royal College of Surgeons of Edinburgh Country: United Kingdom	17	10
8.	Plastic Surgery	Fellowship of the Royal College of Surgeons of Glasgow in Plastic Surgery *Listed as Registrable Specialist Qualification as item (19)(f)(i) in Fourth Schedule*	Royal College of Physicians and Surgeons of Glasgow Country: United Kingdom	14	6
9.	Internal Medicine	Membership of the Royal College of Physicians of Ireland *Listed as Registrable Specialist Qualification as item (21)(f)(i) in Fourth Schedule*	Royal College of Physicians of Ireland Country: Ireland	881	553
		Membership of the Royal College of Physicians *Listed as Registrable Specialist Qualification as item (21)(g)(i) in Fourth Schedule*	Royal College of Physicians of United Kingdom Country: United Kingdom		
10.	Emergency Medicine	Fellowship of the Royal College of Emergency Medicine *Listed as Registrable Specialist Qualification as item (22)(c)(i) in Fourth Schedule*	Royal College of Emergency Medicine of United Kingdom Country: United Kingdom	121	173
11.	Family Medicine	International Conjoint Fellowship of the Royal Australian College of General Practitioners *Listed as Registrable Specialist Qualification as item (23)(b)(ii) in Fourth Schedule*	Royal Australian College of General Practitioners Country: Australia	1,184	475
		Membership of the Irish College of General Practitioners *Listed as Registrable Specialist Qualification as item (23)(c)(i) in Fourth Schedule*	Irish College of General Practitioners Country: Ireland	457	184
12.	Psychiatry	Membership of the Royal College of Psychiatrists *Listed as Registrable Specialist Qualification as item (28)(c)(i) in Fourth Schedule*	Royal College of Psychiatrists of United Kingdom Country: United Kingdom	61	169
13.	Clinical Radiology	Fellowship of the Royal College of Radiologists *Listed as Registrable Specialist Qualification as item (29)(f)(i) in Fourth Schedule*	Royal College of Radiologists of United Kingdom Country: United Kingdom	66	87
14.	Urology	Fellowship of the Royal College of Surgeons of Glasgow in Urology *Listed as Registrable Specialist Qualification as item (30)(d)(ii) in Fourth Schedule*	Royal College of Physicians and Surgeons of Glasgow Country: United Kingdom	34	21

Parallel Pathway Specialist Training Programmes are governed through a central committee in the MOH, and certification of completion of training is issued by the MOH as a training provider; NA = not available
